# Beyond a Dichotomous View of the Concepts of ‘Sex’ and ‘Gender’ Focus Group Discussions among Gender Researchers at a Medical Faculty

**DOI:** 10.1371/journal.pone.0050275

**Published:** 2012-11-20

**Authors:** Lena Alex, Anncristine Fjellman Wiklund, Berit Lundman, Monica Christianson, Anne Hammarström

**Affiliations:** 1 Department of Nursing, Umeå University, Umeå, Sweden; 2 Department of Community Medicine and Rehabilitation, Physiotherapy, Umeå University, Umeå, Sweden; 3 Research Programme Challenging Gender, Umeå University, Umeå, Sweden; 4 Department of Public Health and Clinical Medicine, Umeå University, Umeå, Sweden; 5 Umeå Centre for Gender Studies in Medicine, Umeå University. Research Programme Challenging Gender, Umeå University, Umeå, Sweden; The University of New South Wales, Australia

## Abstract

**Introduction:**

The concepts of ‘sex’ and ‘gender’ are both of vital importance in medicine and health sciences. However, the meaning of these concepts has seldom been discussed in the medical literature. The aim of this study was to explore what the concepts of ‘sex’ and ‘gender’ meant for gender researchers based in a medical faculty.

**Methods:**

Sixteen researchers took part in focus group discussions. The analysis was performed in several steps. The participating researchers read the text and discussed ideas for analysis in national and international workshops. The data were analysed using qualitative content analysis. The authors performed independent preliminary analyses, which were further developed and intensively discussed between the authors.

**Results:**

The analysis of meanings of the concepts of ‘sex’ and ‘gender’ for gender researchers based in a medical faculty resulted in three categories; “Sex as more than biology”, with the subcategories ‘sex’ is not simply biological, ‘sex’ as classification, and ‘sex’ as fluid and changeable; ”Gender as a multiplicity of power-related constructions”, with the subcategories: ‘gender’ as constructions, ‘gender’ power dimensions, and ‘gender’ as doing femininities and masculinities; “Sex and gender as interwoven”, with the subcategories: ‘sex’ and ‘gender’ as inseparable and embodying ‘sex’ and ‘gender’.

**Conclusions:**

Gender researchers within medicine pointed out the importance of looking beyond a dichotomous view of the concepts of ‘sex’ and ‘gender’. The perception of the concepts was that ‘sex’ and ‘gender’ were intertwined. Further research is needed to explore how ‘sex’ and ‘gender’ interact.

## Introduction

Most medical and health researchers acknowledge that both social/cultural and biological factors are important for men’s and women’s health status. For example, women are diagnosed with depression twice as often as men in most Western countries [1], and major reviews conclude that this cannot be explained by biological factors alone [2]–[3]. Also, Anne Fausto-Sterling [4] has developed a model for understanding how social and cultural factors interact with bodily processes by demonstrating how our skeletons interact through biological bodily processes with surrounding social and cultural events from birth throughout our lives in the creation of bone density. These are two of many medical examples which indicate that the concepts ‘sex’ and ‘gender’ are both important in medicine. Even so, the meaning of the concepts has seldom been discussed in the medical literature.

In fact – as shown by a publication from the US National Institute of Medicine (NIM) from the *Committee on Understanding the Biology of Sex and Gender Differences* in 2001 – the concepts of ‘sex’ and ‘gender’ have been used in an inconsistent and confusing way in the scientific literature [5]. The NIM report provided inspiration for “gender-specific medicine” – a research field which defines itself as “the science of how men and women differ in their normal human function and the experience of disease” [6].

King [7], after analysing how the concepts of ‘sex’ and ‘gender’ are employed in biomedical publications, concluded that they are misused. ‘Gender’, for example, is not appropriate to use in an anatomical examination such as a urine test and an autopsy, or in relation to non-human animals, which he says do not display ‘gender’ differences. Further, King argues that the concept of ‘sex’ is not a dichotomous classification because there are a large number of intersexual individuals. Also, a recent analysis of the usage of the concepts of ‘sex’ and ‘gender’ in articles in two journals on gender-specific medicine showed that – in spite of the focus on ‘gender’ in the journal titles – there was a conceptual muddle in the use of ‘sex’ and ‘gender’ [8]. The analyses showed that many of the papers in the journals were ‘sex’- and ‘gender’-blind, i.e. did not even use the concepts of ‘sex’/‘gender’, while most of the other papers used them interchangeably without definitions.

Biological settings still tend to privilege a positivist understanding of the body, including one which invests credibility in the stereotyping of gender roles. However, the concept of gender has been visualised and problematized in medical and health research by gender researchers skilled both according to biological and positivist perspectives (about the body) and to humanist constructive perspectives (about the gender). We define gender researcher as researchers with special interest within the field of gender perspectives in health related research. In this study we mean that gender researchers in medicine and health science, with knowledge both in medicine and gender, can be viewed as “medical insiders”.

In order to provide an understanding of what the concepts of ‘sex’ and ‘gender’ can mean in medicine and in health sciences, this study aimed to explore what the concepts of ‘sex’ and ‘gender’ meant for gender researchers based in a medical faculty.

## Methods

### Setting

The research programme Challenging Gender was financed by the Swedish Research Council during the period 2007–2011 as an interdisciplinary Centre of Gender Excellence at Umeå University. The research programme consisted of five research streams located at different faculties. One of them – Challenging Health – was located at the Medical Faculty. Challenging Health functioned as a platform for collaboration between researchers from several disciplines based in a Medical Faculty, such as family medicine, nursing, midwifery, physiotherapy, public health, rehabilitation medicine and sports medicine. The programme was open for all researchers with an interest of developing gender perspectives in their health- related research. During the five-year period, 20 researchers met regularly in Challenging Health in order to develop gender perspectives and theories in their research. The researchers brought their own ongoing studies to the programme and also planned and performed new studies together.

### Participants

All 20 researchers who participated in Challenging Health were invited to participate in the study, and 16 accepted. All the participants were women and gender researchers with the following positions: two professors, eight senior researchers and six postgraduate students.

### Design

The design of the study, as well as the main questions to be discussed, were decided in advance as part of the Challenging Health project. An interview guide was outlined, based on four themes formulated as questions: *What do the concepts of ‘sex’ and ‘gender’ mean to you? How do you use these concepts in your research? What problems do you meet in your research? How do you solve those problems?* In this paper the first theme (*What do the concepts of ‘sex’ and ‘gender’ mean to you?)* was in focus. The last two themes have been reported elsewhere [9].

### Performance

We decided that focus group discussions were the most appropriate method for exploring what the concepts of ‘sex’ and ‘gender’ mean for the participants. Therefore, during spring 2008 two focus group discussions – each consisting of eight participants – were held, inspired by focus group research principles [10]. This form of qualitative research construes participants as active collaborators in generating knowledge. Researchers from various research areas (general practice, nursing, physiotherapy, public health, rehabilitation medicine and sports medicine) and with various positions were mixed in the two focus groups. The discussions lasted for 90 minutes, were tape-recorded and transcribed verbatim.

The moderators led the focus group discussions by introducing the questions and were actively involved, together with the observer, in managing the group dynamics with the aim of creating possibilities for all participants to make themselves heard in the group [10]. The moderator and the observer encouraged a conversational discussion tone, in order to enable the interviewees to talk more freely. The focus group discussions as well as the analysis were conducted in Swedish and thereafter translated into English.

According to the Regional Ethics Vetting Board in Umeå, Sweden, and the current Swedish legislation about research involving humans [11], this study was exempt from formal ethical review.

All participants in Challenging Health were invited to take part in the focus group discussions. The participants took part on a voluntary basis with verbal informed consent. All who participated in the focus group discussions, including the moderators and the observers, were invited to participate in the analysis and writing of the manuscript. Five of the participants (moderator MC, observer BL and participants LA, AFW and AH) were willing to engage in the analysis of the tape-recorded transcribed focus group discussions and in the manuscript writing. During the analysis and the interpretation of the text all 16 participants in the focus group discussions were invited to various seminars about the analysis.

### Analyses

The focus group discussions were analysed by means of qualitative content analysis as described by Graneheim and Lundman [12]. Content analysis is a method of analysing written or verbal communication in a systematic way [13]. The method is useful in analyses of persons’ or groups’ experiences, reflections, and attitudes [14].

The analysis was performed in several steps. The text was divided into meaning units, each comprised of several words, sentences, or paragraphs containing aspects related to each other through their content and context. Taking the context into consideration, the meaning units were condensed and each was labelled with a code. The codes were interpreted and compared for differences and similarities. In the next step the codes were formulated into preliminary subcategories and categories, which after more discussions, both in an extended research group and in international collaboration, were formulated into three categories and eight subcategories. The subcategories and categories are given in Table 1. All authors (LA, AFW, BL, MC, AH) performed independent analyses of the whole text which were thereafter rigorously discussed in the research group. In this way – in order to increase credibility – triangulation between the authors was achieved [15]. The focus group discussions provided rich data which strengthen our results.

**Table 1:** Identified categories and subcategories.

Categories	Subcategories
“Sex as more than biology”	*‘sex’ is not simply biology*
	*‘sex’ as classification*
	*‘sex’ as fluid and changeable*
“Gender as multiplicity of power-related constructions”	*‘gender’ as constructions*
	*‘gender’ power dimensions*
	*‘gender’ as doing femininities and masculinities*
“Sex and gender as interwoven”	*‘sex’ and ‘gender’ as inseparable*
	*embodying ‘sex’ and ‘gender’*

## Results

The analysis of meanings of the concepts of ‘sex’ and ‘gender’ for gender researchers based in a medical faculty resulted in three categories; **“Sex as more than biology”**, with the subcategories: ‘sex’ is not simply biological, ‘sex’ as classification, and ‘sex’ as fluid and changeable; **“Gender as a multiplicity of power-related constructions”**, with the subcategories: ‘gender’ as constructions, ‘gender’ power dimensions, and ‘gender’ as doing femininities and masculinities; **“Sex and gender as interwoven”**, with the subcategories: ‘sex’ and ‘gender’ as inseparable, and embodying ‘sex’ and ‘gender’.

### “Sex as More than Biology”

The concept of ‘sex’ was discussed in relation to biology and classification as well as by seeing sex as fluid and changeable.

#### ‘Sex’ is not simply biological

The participants thought that the traditional meaning of ‘sex’ was related to biological differences between men and women, such as the outer ‘sex’ characteristics and reproductive organs, reproductive functions, gonad hormones as well as genetic differences. *“There is no body without a sex.”*

A question that was discussed was whether ‘sex’ should be restricted to reproductive differences between men and women or extended to include all biological differences between men and women. Here, the expression from “gender-specific medicine” – “There is sex in every cell” – was discussed. One opinion was that there are “*sex-specific mitochondria in every cell, even in the skin. You can trace women’s mitochondrial DNA a long way back*”, and that *“There is not a disease which is not sex-linked.”* However, the “sex in every cell” slogan was criticized: *“If there are blood pressure differences between men and women, is that sex? Is all biology sex?”.*

Therefore, not all biological differences between men and women could necessarily be regarded as sex-related. Instead, ‘sex’ was seen as related to reproductive differences between men and women while other biological conditions – e.g. hypertension – do not necessarily have anything to do with ‘sex’. In line with this definition, ‘sex’ could mean “*the ability to become pregnant.”*

Biological functioning could be one way of thinking about ‘sex’: “we use joint flexibility or muscle strength – often I think about these as sex even though I do not think about them as unchangeable. But I believe that there is a biological function which I think about as sex.”

This quotation illustrates that ‘sex’ and biology could be seen as changeable.

### ‘Sex’ as Classification

‘Sex’ was described as a common way of categorizing men and women into two separate biological groups, a categorization which starts at birth and continues throughout life. An important linguistic difference – with a broader meaning of ‘sex’ in Swedish compared to English – was discussed for example in relation to questionnaires. As one participant said, *when constructing a questionnaire in Swedish you ask people about their ‘sex’, not about their ‘gender’*. Also, in the Swedish language the concept of ‘sex’ was seen as useful in relation to the categorization of men and women in quantitative research. In a similar way, the participants’ could see benefits in using ‘sex’ rather than ‘gender’ also in English in quantitative research when no or few socio-cultural aspects are included.

The common dichotomous classification of ‘sex’ into men and women was questioned, as it excluded those who do not define themselves in either of these two categories – as men or women. For example, in sports there are only two sexes. *“There is nothing in-between. Even if there is, people must categorize themselves as the one or the other in order to participate in the sport.”*

### ‘Sex’ as Fluid and Changeable

The participants saw themselves as challenging the traditional view of ‘sex’ as static and unchangeable by regarding biological ‘sex’ as being fluid, changeable, and influenced by social environment. Even though new strands have developed in medical research in order to analyse the interactions between biology and culture, one participant was critical and argued that ‘sex’ and biology still dominated and that the possible influence of ‘gender’ on ‘sex’ was seldom analysed.

Thus, the participant criticized medical research and said that biological processes should not be seen as the main or primary starting point in influencing the social environment, but rather as part of the interaction between the social and the biological. In this way, biology was not seen as static but as a response to social processes and as something that can be changed. To inscribe ‘gender’ in biology, instead of the contrary, was seen as a challenging but difficult task for gender researchers in medicine in their understanding of the meaning of the concepts of ‘sex’ and ‘gender’. An example was that it seemed almost impossible to study biological differences without taking gender into account because *“all bodies are influenced by culture and by expectations.”* A similar statement was that: “*The human being is more than biology.*”

The participants discussed how the meaning of ‘sex’ seemed to differ in English and in Swedish. ‘Sex’ as more than biology was expressed as being obvious in Swedish: *“we already have gender in the sex concept which is not the case in English … it is much easier to say sex-segregated rather than gender-segregated labour market …”.*

### “Gender as a Multiplicity of Power-related Constructions”

#### ‘Gender’ as constructions

The concept of ‘gender’ was described as *a socio-cultural construction.* The strong focus on constructivist perspectives was exemplified as: *“Isn’t everything gender?”* ‘Gender’ was seen as strongly influenced by living circumstances, behaviours and attitudes in both society and culture. Using the term ‘gender’ in medicine made it possible to extend biology, as expressed by one researcher: *“As a gender researcher I don’t focus on biological sex, I think in the form of gender processes and social processes.”*

#### ‘Gender’ power dimensions

Power perspectives connected to ‘gender’ were stressed as important to visualize, because women were seen as *“always subordinate to men in some respect…”* Power was described as *“an undercurrent to everything.”* The concept of power was also problematized regarding the domination of white middle-class feminists’ gender theories in gender research, which were questioned as hegemonic in relation to worldwide feminist theories expressed as *“a feminism that makes black women’s problems invisible.”* The concept of hegemony in relation to femininities was discussed from various perspectives and expressed by one researcher as follows: *“You can say that one woman nearly can belong to the hegemonic masculinity”*, implying that there are women who have power and behave like men. Another view of hegemony within femininities was expressed as the idealized expressions of being a female, as shown in commercial advertising stressing the importance of appearance, youth, and slimness. There were also statements stressing that *“the concept of hegemony does not exist among women.”* Power dimensions were also emphasized when visualizing perspectives such as race, education and class.

#### ‘Gender’ as doing femininities and masculinities

‘Gender’ was described as *“the socio-cultural screen that we call femininity and masculinity”*, followed by discussions of various masculinities and femininities. The concepts of femininities and masculinities were seen as ways of constructing oneself as individuals, as women and men and as such were seen as heavily influenced by social constructivist perspectives. *“As a woman you can create various femininities and as a man you can create various masculinities.”* There were also statements revealing the view that *“One masculinity and one femininity can be nearly the same.”* The researchers were reflecting on how femininities and masculinities were not connected to being a woman or a man: *“You can be a biological man and have a social femininity.”* Other statements were*: “It doesn’t work if only women create femininities!”* and *“I leave it open that men can create femininities.”* Thus, in spite of having varying opinions it was seen as important for gender researchers to contextualize these concepts.

### “Sex and Gender as Interwoven”

#### ‘Sex’ and ‘gender’ as inseparable

The focus group discussions showed that the participants mainly regarded ‘sex’ as biology and ‘gender’ as constructions. However, they also saw the concepts of ‘sex’ and ‘gender’ as co-existing and interactive. *“Sex cannot exist without gender and gender cannot exist without sex.”* The concepts were seen as always linked together and hard to differentiate from each other: *“You can’t speak of sex and gender as separate.”* However, in various contexts, the participants thought that there could be more focus on ‘sex’ or ‘gender’.

The interplay between ‘sex’ and ‘gender’ was described, for example, with the metaphor of a “Möbius band”, always linked together, like a loop with no end. *“The outside of the Möbius band is related to as biology/sex and if you follow the band, suddenly you are on the inside of the band and it has become gender…you follow the band and end up on the outside again and gender has gone back to biology.”*

A new ‘gender’ concept was suggested to be “biological sex with a holistic perspective.” With a new concept, there could be more focus on questions of ‘gender’. “Gender must be added when you look at biological sex… it becomes biological sex covered with social factors (such as power dimensions, financial circumstances etc.).”

#### Embodying ‘sex’ and ‘gender’

The body was seen as important in relation to the concepts of ‘sex’ and ‘gender’ since the body makes ‘gender’. The concept of embodiment was held up as useful for bridging the dichotomies between ‘sex’ and ‘gender’ and as a way of discussing ‘sex’, ‘gender’ and bodies. Embodiment was described as *“how the body interacts with and reacts to the environment we live in and to society”* and viewed as a concept which *“really goes into the body; the body interacts with the environment through embodiment and the body is influenced by the lived experience.”* A participant gave the example of how repeated sexual abuse can change the body and the abuse can become manifest in the body and is visible as a changed posture. Post-traumatic stress disorders and refugees with paradoxical bodily reactions could also serve as examples of how the body can react to the environment and to certain experiences. Other participants were hesitant about using the concept of embodiment since *“it has often been used in relation to ‘the surface of the body’ such as muscles and body-shape and has seldom been used in relation to gendered biological processes and life itself.”*

## Discussion

### On the Results

The analysis of what the concepts of ‘sex’ and ‘gender’ mean for gender researchers based in a medical faculty resulted in three categories; **“Sex as more than biology”**, with the subcategories: *‘sex’ is not simply biological*, *‘sex’ as classification*, and *‘sex’ as fluid and changeable*; **”Gender as a multiplicity of power related constructions”**, with the subcategories: ‘*gender’ as constructions, ‘gender’ power dimensions*, and *‘gender’ as doing femininities and masculinities*; and **“Sex and gender as interwoven”**, with the two subcategories: *‘sex’ and ‘gender’ as inseparable* and *embodying ‘sex’ and ‘gender’*. The study showed that the concepts of ‘sex’ and ‘gender’ were complicated and hard to define, even among researchers with quite extensive experience in gender issues in health.

The gender researchers in this study thought that the traditional definition of ‘sex’ was not sufficient for understanding the biological body. Instead they stressed that it was difficult to define where ‘sex’ and ‘gender’ started and ended as the concepts were seen as intertwined. Social, cultural and historical influences were seen as important in relation to bodily symptoms and medical diagnosis and prognosis [16]–[17]. Thus, the focus groups’ apprehension of the concept of ‘sex’ was that it was not static and unchangeable; instead they stressed that ‘sex’ as biology was influenced by culture and society. This perspective is in accordance with research by biological feminist researchers such as Birke [18], who reject the assumption of the view of biology as fixed and unchangeable, claiming that biological science offers more than a deterministic view of how the body works. Also, Fausto-Sterling [4] introduced a life-course systems approach to analyses of ‘sex’ and ‘gender’, focusing on the interweaving of the two concepts throughout the life span. Bird and Rieker [19] stress that researchers must study both biological and social factors simultaneously.

The focus group discussions revealed that the participants in our study looked for new ways of understanding ‘sex’ and ‘gender’, beyond the traditional concepts. The participants emphasized the body as important in relation to ‘sex’ and ‘gender’, and the concept of embodiment was suggested as a possible bridge between these concepts. Krieger [20]–[21], in her eco-social theory, refers to embodiment as an idea of how we biologically incorporate the world we live in, including our societal and ecological circumstances. According to her theory, embodiment is a concept interacting between bodies, components of bodies, and the world in which the bodies live. No aspect of our biology can be understood without knowledge of history and of societal and individual ways of living. She argues that embodiment can be used for analyses from the micro level to a system level. The eco-social theory includes more than adding biological factors to a social analysis, or social factors to a biological analysis. This theory has a systematic integrated approach.

To deal with the interaction between the concepts of ‘sex’ and ‘gender’ in a slightly different manner, Öhman Hägg and Dahlgren [22] have proposed that “men and women are neither ‘sex’ nor ‘gender’, but biological, social and cultural beings in complicated and changing combinations and power relations”. They criticize health-care professions for being rather gender-blind and not focusing sufficiently on gendered power relations, for example, in health care.

Our study showed that ‘gender’ was seen as power-related constructions, which can be compared to the definition by Simone de Beauvoir [23] who wrote: *“We are not born women, we become women”*, indicating how social, cultural and symbolic influences contribute to constructions of femininities and masculinities. Power dynamics were mentioned in various ways in the focus group discussions. The gender order, male dominance, and male preferential right of interpretation were stressed [16], [23]–[25]. Power was also visualized as hegemonic masculinity, which was seen as a useful and comprehensible theoretical concept. Hegemonic masculinity has been problematized and researchers have illuminated constructions of various masculinities in relation to health [24], [26]–[27] and ageing [28]. There were few considerations about hegemonic masculinity, but the possibility of a hegemonic femininity was questioned.

The constructivist view in our study contrasts with the common view in medicine of gender as stereotypical role behaviour [29], focusing on harmony between men and women. Role theories have been criticized by feminist medical researchers for assuming a harmony between men and women which discounts gendered conflicts [29].The theories have also been criticized for being too simplistic in describing men but especially women as passive objects of social norms. Thus, the participants in our study were in the forefront of gender research also in relation to the meaning they gave to the concept of ‘gender’.

Gender-constructive theorists, such as Butler [30], have tried to transcend the body to understand how gender is constructed. However, our study showed that for a gender researcher in medical sciences it seems impossible to transcend the body in the field of medicine, because in practice the health care workers meet the body, which wants to be cured and cared for. Just looking at the human being as historically, culturally and socially constructed and leaving the body out of account was seen as problematic.

The first and second categories might be seen to represent the 'positivist' and 'constructionist' understandings of gender, but the third category begins to weave these together. This implies that gender researchers working in health and medical settings try to find useful ways to draw upon both biomedical and sociological understandings of the relationship between the body and society. The third category with its focus on “Sex and gender as interwoven” is in line with the first category (“Sex as more than biology”) but may seem to contradict the second category (“Gender as power-related constructions”) which does not include any biological perspectives. A reason could be that gender theories have mainly been developed within the social sciences with few references to the body and the biological [16]. But in order to discuss how ‘sex’ and ‘gender’ are interwoven we think that we first need to define each concept. Therefore, the results in relation to the third category may be viewed as an extension of the first two categories.

### On the Methods

In order to collect complete data, the focus group discussions were audio-taped and transcribed. The focus group participants represented a broad spectrum of disciplines in medicine as well as a variation in academic knowledge, from novices in the field of gender studies to very experienced gender researchers. We believe that the variation in disciplines and academic knowledge has contributed both to a more varied discussion and to a broader analysis of the focus group discussions.

#### Limitations

A possible risk of having both doctoral students and professors in the focus groups may have been inequity of power between the participants. In order to handle the possible imbalance, the moderators/observers encouraged all participants to engage in the discussions. Another limitation could be that the study only included two focus groups. However, all gender researchers based in a medical faculty were invited and the intention was to have focus groups including enough participants to capture variation. No men participated because at that time only women were part of the research group. In a Scandinavian (and also worldwide) context the majority of gender researchers are women, but more and more men are entering the research field, and our results could have been more varied if also men had participated.

We, as authors, both participated in the focus groups and analysed the data. This is not quite in accordance with traditional focus group analysis. However, we found this form of qualitative analysis fruitful and challenging. As researchers, both participating and analysing may have led to over-interpretation of the data. To handle this problem, coding and categorization were first done independently, followed by constant discussions between the authors regarding the outcome of the analysis to achieve a negotiated outcome [15]. The outcome was further discussed in extended national and international research groups.

### Conclusions

Gender researchers working in the fields of medicine and health stressed the importance of looking beyond a dichotomous view of the concepts of ‘sex’ and ‘gender’. The concept of ‘sex’ was seen to operate as biology and more while the concept of gender was seen to be produced as a multiplicity of power-related constructions. Further research is needed to explore how ‘sex’ and ‘gender’ are articulated in relation to each other, and provide a mechanism whereby gender researchers can draw upon both positivist and constructionist philosophies of the body in society. This study adds the specific views of gender researchers in medicine and health sciences – “medical insiders” – to bring new challenges into focus.
